# A Sustainable Approach for Degradation of Alternariol by Peroxidase Extracted from Soybean Hulls: Performance, Pathway, and Toxicity Evaluation

**DOI:** 10.3390/foods13152434

**Published:** 2024-08-01

**Authors:** Xingke Zhang, Hao Zheng, Hao Lv, Jiyuan Yin, Yi Li, Kexin Zhang, Liangyu Zhang, Wei Zhang, Zhixiang Wang, Lihong Zhao, Yongpeng Guo

**Affiliations:** 1 College of Animal Science and Technology, Henan Agricultural University, Zhengzhou 450046, China; 18239791502@163.com (X.Z.); april262022@163.com (H.Z.); lcxhau@163.com (H.L.); 13569779776@163.com (J.Y.); iyizz123@163.com (Y.L.); m19503882026@163.com (K.Z.); zhly0371@163.com (L.Z.); weizhang@henau.edu.cn (W.Z.); wzxhau@henau.edu.cn (Z.W.); 2 College of Animal Science and Technology, China Agricultural University, Beijing 100193, China; zhaolihongcau@cau.edu.cn

**Keywords:** soybean hull peroxidase, alternariol, biodegradation, degradation pathway, toxicity evaluation

## Abstract

Alternariol (AOH), an emerging mycotoxin, inevitably exists widely in various food and feed commodities with cereals and fruits being particularly susceptible, raising global concerns over its harm to human and livestock health. The development of eco-friendly and efficient strategies to decontaminate AOH has been an urgent task. This study provided insight into the utilization of crude soybean hull peroxidase as a powerful biocatalyst for degrading AOH. The results confirmed that crude soybean hull peroxidase (SHP) could catalyze the oxidation of AOH by use of H_2_O_2_ as a co-substrate. The optimum reaction conditions for SHP-catalyzed AOH degradation were recorded at pH 4.0–8.0, at 42–57 °C, and at H_2_O_2_ concentration of 100–500 μM. Mass analysis elucidated the degradation of AOH through hydroxylation and methylation by crude SHP. Moreover, toxicological analysis indicated that crude SHP-catalyzed AOH degradation detoxified the hepatotoxicity of this mycotoxin. The performance of crude SHP to degrade AOH in food matrices was further evaluated, and it was found that the enzyme agent could achieve AOH degradation by 77% in wheat flour, 84% in corn flour, 34% in grape juice, and 26% in apple juice. Collectively, these findings establish crude SHP as a promising candidate for effective AOH degradation, with potential applications in the food and feed industry.

## 1. Introduction

The global issue of mycotoxin contamination in food and feed presents a serious threat to human and livestock health and brings huge economic losses to the international trade. Alternariol (AOH), a toxic secondary metabolite mainly produced by some *Alternaria* fungi, is classified as an emerging mycotoxin [1]. AOH contamination has been detected in a range of agricultural commodities, such as cereals, oilseeds, fruits, and their derived products [2]. Occurrence data for AOH in food and feed in Europe showed that 31% of samples were contaminated by this mycotoxin, with elevated levels found in samples exhibiting visible signs of *Alternaria* rot infection [3]. In China, AOH was detected in 38.3% of urine samples of the population living in the Yangtze River Delta [4]. AOH has been reported to be a potent mutagen that can induce DNA damage and chromosomal aberration in mammalian cell lines [5,6]. Chronic exposure to AOH may be a contributing factor in the pathogenesis of esophageal carcinoma [7]. AOH displays chemical structure similarities to estrogen-mimicking substances like urolithin and genistein, and in this regard its endocrine-disrupting effects have also been documented [8,9]. Considering the potential mutagenicity and estrogenic toxicity of AOH, the European Food Safety Authority (EFSA) has established the threshold of toxicological concern (TTC) value for AOH at 2.5 ng kg^−1^ body weight per day [10].

AOH is relatively stable during general food processing operations and storage. Estiarte et al. [11] investigated the influence of heat treatment on the AOH stability in tomato products, and observed that only 33% of AOH was destroyed after 90 min of heating at 100 °C. Moreover, Elhariry et al. [12] described that AOH remained constant in fruit juice after pasteurization. The development of safe, efficient, and sustainable approaches for reducing AOH contamination has been considered an urgent issue to ensure food and feed safety. To date, research on AOH degradation mainly focuses on advanced oxidation processes. Wang et al. [13] reported that dielectric barrier discharge cold plasma could achieve 100% AOH degradation, but the AOH degradation pathway was not characterized. A recent report by Qi et al. [14] showed that pulsed light treatment could break the lactone bond in AOH, leading to a significant reduction of its hepatotoxicity. However, the commercial application of advanced oxidation processes for eliminating AOH in food and feed is hindered by the lack of equipment that can work at high capacity and continuous mode.

In the past few years, there has been a growing interest in the enzymatic degradation of mycotoxins due to its high efficiency, mild reaction conditions, and lack of secondary pollution. Several mycotoxin-degrading enzymes such as aflatoxin oxidase (AFO) [15], fumonisin amine oxidase (AnFAO) [16], zearalenone hydrolase (ZHD101) [17], ochratoxin A hydrolase (OTase) [18], and deoxynivalenol dehydrogenase (DDH) [19] have been identified. Moreover, ligninolytic enzymes, including laccases and peroxidases, are highly non-specific and capable of simultaneously degrading multiple mycotoxins owing to their high redox potential. CotA laccase from *Bacillus licheniformis* possesses oxidation activities on aflatoxin B_1_ (AFB_1_), zearalenone (ZEN), and AOH [20,21,22]. Manganese peroxidases (MnPs) have displayed the potential to be used as a simultaneous degradation agent of AFB_1_, ZEN, deoxynivalenol (DON), and fumonisin B1 (FB1) with the help of dicarboxylic acid as a redox mediator [23]. Soybean hull peroxidase (SHP) is present in a significant amount within soybean hulls, a byproduct derived from the soybean processing industry, which is a cheap and abundant peroxidase source. Our previous study has confirmed that SHP can directly catalyze the aromatic hydroxylation of ZEN in the absence of redox mediator [24]. In view of AOH as a di-phenolic compound structurally similar to ZEN, we hypothesize that SHP may function as a potential AOH-degrading enzyme. In the current work, we studied the usefulness of crude SHP extract for degrading AOH. The degradation products were preliminarily identified by ultra-high-performance liquid chromatography high-resolution mass spectrometry analysis, and the toxicity of which were evaluated with human fetal hepatocyte cell line L-02. Moreover, we investigated the AOH-removal performance of crude SHP extract in various food matrices. This study represented the first attempt to apply peroxidase to degrade AOH and could also promote the resource utilization of soybean hulls.

## 2. Materials and Methods

### 2.1. Chemicals and Reagents

Soybean hulls were obtained from Yihai Grain and Oil Industry Co., Ltd. (Yantai, China). 2, 2′-azino-bis-(3-ethylbenzothiazoline-6-sulphonic acid) (ABTS) and AOH were procured from Sigma Aldrich Co., Ltd. (Shanghai, China). The remaining chemicals were provided by Sinopharm Chemical Reagent Co., Ltd. (Beijing, China).

### 2.2. Extraction of SHP from Soybean Hulls and Tested for Its Ability to Degrade AOH

The experimental parameters of ionic strength, temperature, and duration were optimized for the extraction of peroxidase from soybean hulls. Specifically, 1 g of soybean hulls were placed in a 100 mL conical flask with either 20 mL of distilled water or sodium phosphate buffer at varying concentrations (50, 100, 150, and 200 mM). The extraction process was carried out at 37 °C with orbital shaking at 180 rpm for a duration of 2 h. Subsequently, the soybean hull extract underwent centrifugation at 12,000× *g* and 4 °C for 10 min, followed by filtration using a glass microfiber filter. The enzymatic activity of crude soybean hull peroxidase (SHP) was measured with ABTS as the substrate at 37 °C in sodium citrate buffer (100 mM, pH 4.0) in the presence of 100 μM of H_2_O_2_. One unit of peroxidase activity was defined as the quantity of enzyme required to oxidize 1 μM of ABTS per minute. For the optimization of temperature and time for SHP extraction, 1.0 g of soybean hulls was extracted with 20 mL of sodium phosphate buffer (100 mM, pH 7.0) at 27, 37, and 47 °C for 1, 2, 3, 4, 5, and 6 h. To preliminarily assess the AOH-oxidizing capability of crude SHP, a mixture containing 10 μg mL^−1^ of AOH, 1.0 U mL^−1^ of crude SHP, and 100 μM of H_2_O_2_ was incubated at 37 °C for 1 h. Additionally, the control consisting of only crude SHP and AOH was also prepared. Following the reaction, the samples were analyzed using high-performance liquid chromatography (HPLC) according to the method outlined in Section 2.5.

### 2.3. Molecular Docking Study

The 3D structure of SHP was retrieved from the RCSB Protein Data Bank with the PDB ID: 1FHF, subjected to modifications including removal of water, the addition of hydrogens and Gasteiger charges, and then exportation in PDBQT format. The 3D conformation of AOH was sourced from the PubChem database (Compound CID: 5359485). Molecular docking was performed with AutoDock 4.2. The docking simulation was performed through 100 separate genetic algorithms (GA) runs, 2.5 × 10^7^ number of evaluations for Lamarckian GA method. The docking models were organized based on binding free energy, and the conformation with the lowest binding energy was visualized with PyMOL 2.1.

### 2.4. Enzymatic Characterization of AOH Degradation by Crude SHP

The enzymatic characteristics of crude SHP for oxidizing AOH were studied. To investigate the pH effect on AOH degradation, the reaction mixture containing 2.0 U mL^−1^ of crude SHP, 10 μg mL^−1^ of AOH, and 100 μM of H_2_O_2_ was incubated in different pH buffers (pH 3.0 to 10.0) at 37 °C for 1 h. The optimum temperature for crude SHP-catalyzed AOH degradation was evaluated across a temperature range of 22 to 57 °C. Enzyme quantity optimization for the degradation of AOH was conducted within the range of 0.1 to 5.0 U mL^−1^ at 37 °C and pH 7.0. The influence of initial H_2_O_2_ concentration on AOH degradation was studied from 10 to 500 μM at 37 °C and pH 7.0.

### 2.5. Determination of AOH by HPLC

The AOH content was detected by HPLC equipped with a fluorescence detector (LC-10AT, Shimadzu, Tokyo, Japan). The fluorescence detection condition was set at excitation at 250 nm and emission at 350 nm. Separation was achieved using a Diamonsil^®^ C18 reverse phase column (4.6 × 250 mm, 5 µm) with a mobile phase consisting of 2% acetic acid: acetonitrile (40:60, *v*/*v*) at a flow rate of 1 mL min^−1^. The sample loading volume was 20 μL.

### 2.6. Identification of AOH Degradation Products by UHPLC-HRMS

The transformation products of AOH by crude SHP were identified using ultra-high-performance liquid chromatography high-resolution mass spectrometry (UHPLC-HRMS). Chromatographic separation was conducted on an ACQUITY UPLC H-Class PLUS system equipped with an Acquity HSS T3 C18 column (2.1 × 100 mm, 1.7 μm) (Waters Co., Milford, MA, USA). The mobile phase consisted of water with 0.1% formic acid (phase A) and acetonitrile (phase B) at a flow rate of 0.3 mL min^−1^. The gradient elution protocol was as follows: 2% B for 0–1 min; 2–30% B for 1–7 min; 30–80% B for 7–14 min; 80–95% B for 14–17 min; and 95% B for 17–20 min. The sample loading volume was 2 μL. Mass spectrometry analysis was performed using an AB SCIEX TripleTOF 6600+ system (SCIEX, Framingham, MA, USA) equipped with an electrospray ionization (ESI) source operating in the negative ion model. TOF-MS and TOF-MS/MS were scanned in the mass range of *m*/*z* 50–1200. Dynamic background subtraction (DBS) trigger information-dependent acquisition (IDA) was utilized to trigger acquisition of MS/MS information. Data acquisition and processing were performed using Analyst TF™ and MetabolitePilot™ Software 2.0 (AB, Milford, MA, USA).

### 2.7. Cytotoxicity Assessment of AOH Degradation Products

Human fetal hepatocyte cell line L-02 obtained from Tongpai biotechnology Co., Ltd. (Shanghai, China) was cultured in Dulbecco’s Modified Eagle’s Medium (DMEM) containing 10% fetal bovine serum and 100 U mL^−1^ of penicillin and streptomycin, in a humidified incubator with 5% CO_2_ at 37 °C. Cell viability was determined using the Cell Counting Kit-8 (CCK-8, C0038, Beyotime Institute of Biotechnology, Shanghai, China). L-02 cells were seeded in a 96-well plate at a density of 1 × 10^4^ cells each well and exposed to AOH at concentrations ranging from 10 to 100 μM for 24 h. Afterwards, 10 μL of CCK-8 reagent was introduced into each well and incubated at 37 °C for 1 h. The absorbance at 450 nm was detected with a microplate reader to calculate cell viability. To assess the cytotoxicity of AOH degradation products, the viability of L-02 cells exposed to 100 μM of AOH and crude SHP-catalyzed AOH degradation products for 24 h was measured. The lactate dehydrogenase (LDH) activity in the culture supernatant was quantified using a commercial kit (BC0685, Solarbio Technology Co., Ltd., Beijing, China). The content of malondialdehyde (MDA) in the cell homogenate was determined by the thiobarbituric acid (TBA) reaction using a commercial kit (BC0025, Solarbio Technology Co., Ltd.). Moreover, the levels of reactive oxygen species (ROS) and cell apoptosis were also measured. L-02 cells were plated in 6-well plates at a density of 3 × 10^5^ cells per well and exposed to AOH and its degradation products for 24 h. The ROS levels were measured using a Dichlorodihydro-fluorescein Diacetate (DCFH-DA) assay (BL714A, Biosharp, China). Following treatment with AOH and its degradation products, L-02 cells were incubated with 10 µM of DCFH-DA at 37 °C for 30 min, and then were washed three times with PBS. The fluorescence intensity was captured using a fluorescence microscope (Olympus, Tokyo, Japan) and quantified using ImageJ software 1.8.0. Cell apoptosis rate was measured using an Annexin V-fluorescein isothiocyanate (FITC) Apoptosis Detection Kit (BMS500FI-100, eBioscience, San Diego, CA, USA). Briefly, L-02 cells exposed to AOH and its degradation product were dual-stained with Annexin V-fluorescein isothiocyanate (FITC) and propidium iodide (PI), followed by flow cytometric analysis.

### 2.8. Application of Crude SHP to Degrade AOH in Food Matrices

The ability of crude SHP for detoxification of AOH in food matrices was evaluated. The fruit juice degradation system was as follows: 50 mL of fruit juice, 2 U mL^−1^ of SHP, 100 μM of H_2_O_2_, and 1 h reaction at 42 °C. For cereal powder, 10 g of sample with AOH content at 5 μg g^−1^ was suspended in 50 mL of sodium phosphate buffer (100 mM, pH 8.0). Following the addition of 2 U mL^−1^ of SHP and 100 μM of H_2_O_2_, the mixture was incubated at 42 °C for 1 h. Subsequently, a 10 mL aliquot of the mixture was combined with 10 mL of acetonitrile, vortexed for 15 min, and then centrifuged at 12,000× *g* for 10 min. A 2 mL aliquot of the supernatant was mixed with 8 mL of PBS and added to an Alternariol Immunoaffinity Column (IAC-100-3, Pribolab, Qingdao, China) according to the manufacturer’s guidelines. The eluent was subjected to AOH quantification by HPLC as described in Section 2.5.

### 2.9. Statistical Analysis

The data were subjected to statistical analysis through one-way analysis of variance (ANOVA), followed by Tukey’s Honestly Significant Difference (HSD) test, with statistical significance set at *p* < 0.05. Statistical analysis was performed using SPSS V20.0 software, and graphical representations were created using GraphPad Prism 7. The results were reported as mean ± standard deviation (mean ± SD).

## 3. Results and Discussion

### 3.1. Extraction of SHP from Soybean Hulls and Tested for Its Ability to Degrade AOH

**Figure 1:**
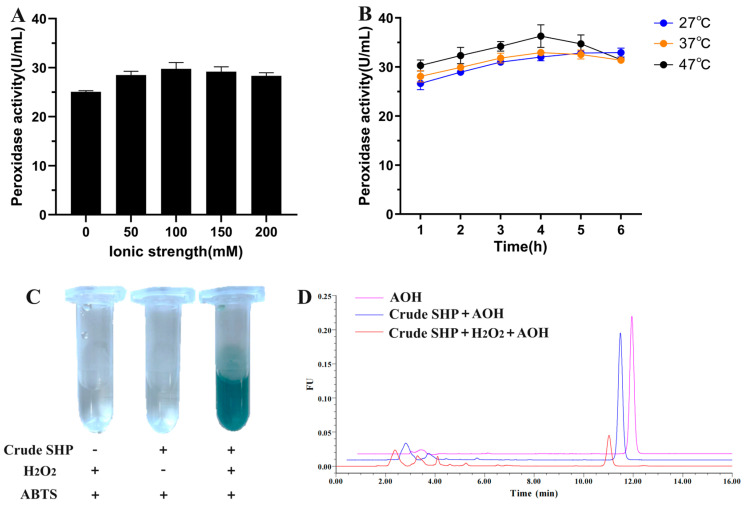
Extraction of crude SHP from soybean hulls and tested for its ability to degrade AOH. (**A**) Effect of ionic strength of extract solution on peroxidase extraction from soybean hulls. (**B**) The time course of peroxidase activity after extraction at 27, 37, and 47 °C. For A and B, the values are mean ± SD of three replicates. (**C**) Photographs for recording the ABTS oxidation reaction by crude SHP extract. “+” and “−” represented presence and absence, respectively. (**D**) HPLC chromatograms of AOH after degradation by crude SHP.

The optimum conditions required for obtaining a high amount of peroxidase from soybean hulls during the extraction process were first investigated. Sodium phosphate buffer (pH 7.0) with different ionic strengths and distilled water were used as solvent to extract SHP from soybean hulls, and the result showed that 100 mM of sodium phosphate buffer yielded the highest peroxidase activity (Figure 1A). In the literature [25], the best outcomes for peroxidase extraction from various plant sources were obtained by using phosphate buffer with ionic strength at 10 to 100 mM and pH 5.0 to 7.0. The extraction temperature and time were also crucial for the release of peroxidase from soybean hulls and avoiding enzyme inactivation during extraction. The extraction process was conducted at 27 °C, 37 °C, and 47 °C for 1 to 6 h, and the maximum peroxidase activity was recorded at 36.3 U mL^−1^ after extraction at 47 °C for 4 h (Figure 1B). The presence of laccase in the SHP extract was also tested, as the soybean genome contained 93 putative laccase genes [26]. Unlike peroxidase, laccase did not require H_2_O_2_ as a co-substrate. The AOH-oxidizing activity of laccase was documented in a previous study [22]. As shown in Figure 1C, the reaction mixture exhibited no color change when it only contained SHP extract and ABTS, which indicated the absence of laccase in the extract. The AOH-oxidizing activity of the extract was further examined by HPLC analysis. The retention time of AOH was 11.0 min, and the peak area of AOH was reduced by about 70% after treatment by crude SHP in the presence of H_2_O_2_ (Figure 1D). On the contrary, AOH could not be degraded by crude SHP extract in the absence of H_2_O_2_. These findings confirmed that the peroxidase in the extract catalyzed the degradation of AOH.

### 3.2. In Silico Analysis of SHP Interaction with AOH

**Figure 2:**
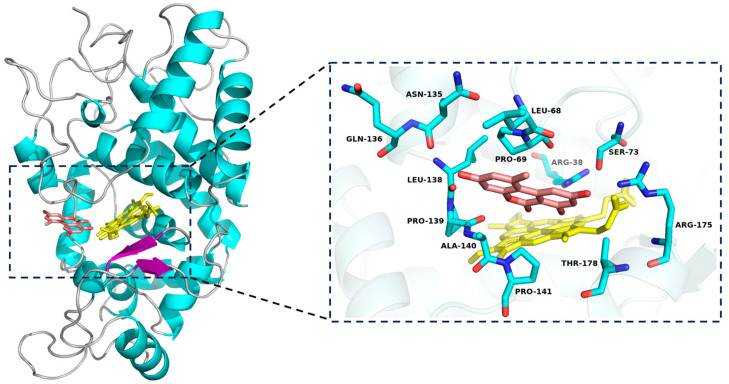
Molecular docking study of SHP with AOH. The left part displayed the AOH-binding pocket of SHP, while the right part showed the detailed interaction. AOH was rendered in salmon, and the heme group of SHP was colored in yellow.

Molecular docking is an effective approach to investigate the binding interactions within enzyme-substrate complexes, which can help to clarify the molecular mechanism of enzyme reaction. The result depicted in Figure 2 showed the successful docking of AOH within a special cavity adjacent to the heme group of SHP, and the binding free energy for the optimal docking model of AOH with SHP was—6.5 kcal/mol. The low binding free energy suggested that AOH had a strong affinity with SHP. The substrate binding pocket was formed by Arg 38, Leu 68, Pro 69, Ser 73, Asn 135, Gln 136, Leu 138, Pro 139, Ala 140, Pro 141, Arg 175, and Thr 178. The binding site for AOH was nearly same as that for ZEN, as the amino acids Leu 68, Pro 69, Ser 73, Leu 138, Pro 139, Ala 140, Pro 141, Arg 175, and Thr 178 were engaged in the binding with ZEN [24]. The result of molecular docking analysis was consistent with experimental data, both indicating that AOH was a suitable substrate for SHP.

### 3.3. Enzymatic Characterization of AOH Degradation by Crude SHP

**Figure 3:**
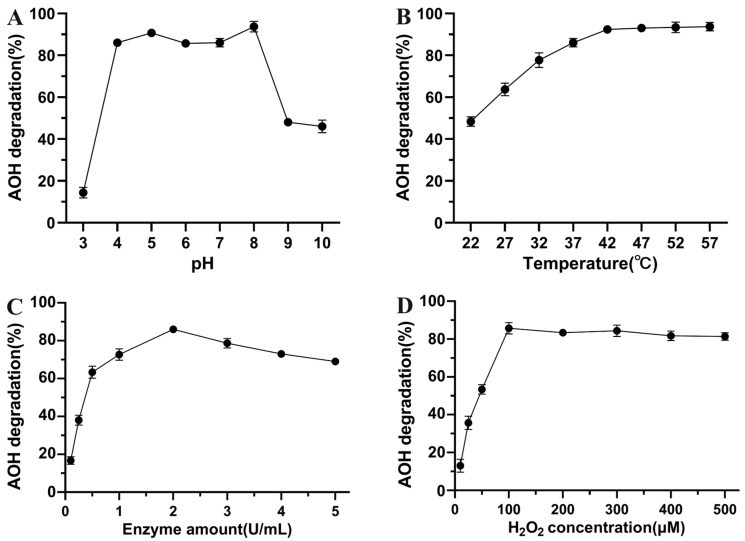
Enzymatic characterization of crude SHP for degrading AOH. Effect of pH (**A**), temperature (**B**), enzyme amount (**C**), and H_2_O_2_ concentration (**D**) on crude SHP-catalyzed AOH degradation. Data are mean ± SD, *n* = 3.

The influence of pH, temperature, enzyme quantity, and H_2_O_2_ concentration on AOH degradation by crude SHP was investigated. The optimum pH range of crude SHP for AOH degradation was found to be between pH 4.0 to 8.0 with the degradation rate maintaining above 85%. The highest rate of AOH degradation, reaching 94%, was achieved at pH 8.0 (Figure 3A). Similarly, SHP was previously found to display the maximum activity towards ZEN at pH 8.0 but failed to oxidize ZEN at pH 4.0 [24]. Moreover, commercial horseradish peroxidase (HRP) achieved the highest ZEN and ochratoxin A (OTA) degradation at pH 6.0 to 8.0 [27]. By contrast, the optimum pH was recorded at pH 5.5 for DON degradation by rice bran peroxidase (RBP) [28]. MnP from *Phanerochaete chrysosporium* and *Irpex lacteus* were active at pH 4.0 to 5.5 for oxidizing AFB_1_, ZEN, and DON, and dramatically lost their mycotoxins-degrading activity in strongly acidic and neutral conditions [29]. The optimum temperature of plant peroxidases varies greatly with the enzyme source, ranging from 16 °C for vanilla bean peroxidase (VBP) [30] to 60 °C for elephant foot yam corm peroxidase (ECP) [31]. In this study, the degradation rate of AOH increased linearly from 48% at 22 °C to 92% at 42 °C, and then plateaued as temperature further increased up to 57 °C (Figure 3B). Taken together, crude SHP had excellent AOH-oxidizing activity across a broad range of pH and temperature. Enzyme amount was also an important parameter for the optimization of crude SHP-catalyzed AOH degradation. The AOH-degradation rate increased sharply from 17% to 63% with the addition of crude SHP from 0.1 to 0.5 U mL^−1^, gradually ascended to 86% at 2.0 U mL^−1^, and then gradually decreased to 69% at 5.0 U mL^−1^ (Figure 3C). The degradation curve was in good agreement with that in the process of ZEN degradation by SHP, in which the maximum degradation rate was obtained at a SHP amount of 2.0 U mL^−1^, and the presence of a higher SHP resulted in a reduction of the ZEN degradation rate [24]. Likewise, Marimón Sibaja et al. [32] reported that the degradation of AFB_1_ by commercial HRP declined from 69% to 26% as the enzyme amount increased from 0.015 to 1.0 U mL^−1^. These results might be explained by the quick decomposition of H_2_O_2_ into H_2_O and O_2_ by the excess peroxidases, which will no longer be available to act as an electron acceptor for substrate oxidation. Conversely, high concentrations of H_2_O_2_ may lead to enzyme inactivation. Thus, the influence of H_2_O_2_ concentration on AOH degradation was investigated. As shown in Figure 3D, the AOH degradation rate exhibited a pronounced acceleration upon the introduction of H_2_O_2_ from 10 to 100 μM, and then plateaued as the initial H_2_O_2_ was further raised up to 500 μM. The result was consistent with our previous report, which suggested that the optimal degradation of ZEN occurred at an H_2_O_2_ concentration of 100 μM [24]. The similar behavior was observed by Fernandes et al. [33] using SHP for the removal of 2,4-dichlorophenol.

### 3.4. Identification of AOH Degradation Products

**Figure 4:**
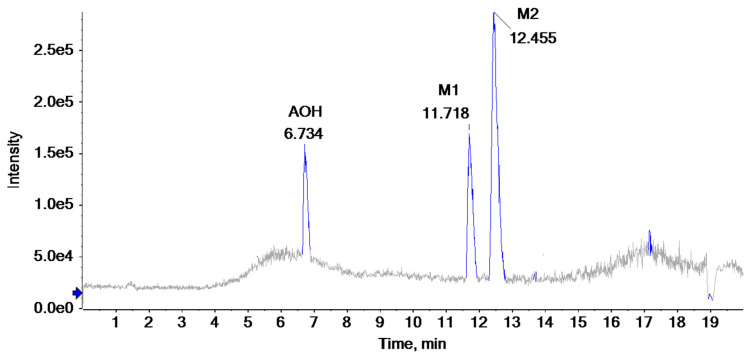
Total ion chromatogram of degradation products following degradation of AOH by crude SHP.

To clarify the AOH degradation pathway catalyzed by crude SHP and to understand the degradation mechanism, UHPLC-HRMS analysis was conducted to identify the AOH degradation products. AOH displayed the parent ion at *m*/*z* 257.0459 and generated fragment ions of *m*/*z* 215.0385 and *m*/*z* 147.0477 (Figure S1 and Table 1). As depicted in the total ion chromatogram (Figure 4), the degradation of AOH resulted in the formation of two transformation products named M1 and M2. Detailed information about the retention time, formula, accurate mass, fragment ions, and inferred chemical structure of the two degradation products were summarized in Table 1. The two products were formed by aromatic hydroxylation and subsequent methylation. Similarly, Sun et al. [22] also documented hydroxylation of AOH catalyzed by CotA laccase. Additional research is required to purify the degradation products and elucidate their chemical structure through the use of nuclear magnetic resonance (NMR).

### 3.5. Cytotoxicity Evaluation of AOH Degradation Products

**Figure 5:**
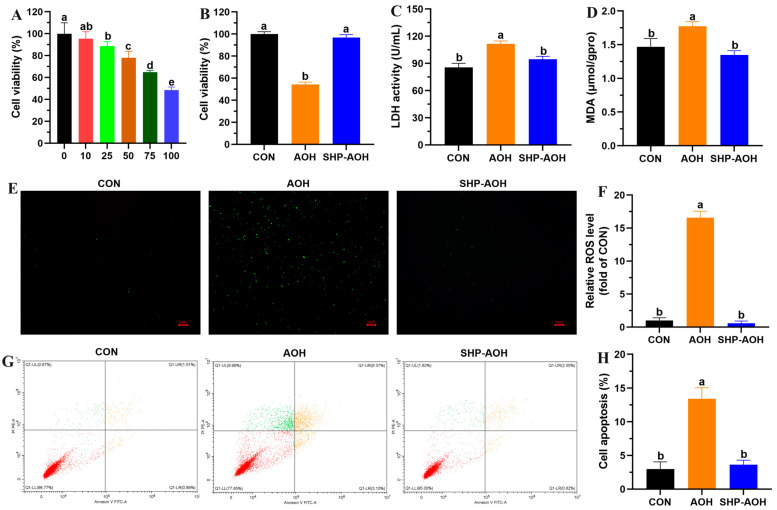
Cytotoxicity evaluation of AOH and crude SHP-catalyzed AOH degradation products. (**A**) The viability of L-02 cells exposed to various concentrations of AOH. (**B**) The viability of L-02 cells exposed to AOH and crude SHP-catalyzed AOH degradation products. For A and B, data are mean ± SD, *n* = 6. (**C**) LDH activity. (**D**) MDA content. (**E**,**F**) ROS level. The fluorescence intensity of the cells stained with DCFH-DA was captured by fluorescence microscope. (**G**,**H**) Cell apoptosis rate. The dual-stained cells were depicted by flow cytometry. For C to H, data are mean ± SD, *n* = 3. Different letters indicate significant difference between groups (*p* < 0.05).

The liver, being the primary site for AOH metabolism, is also the organ most affected by AOH pathology. In this study, the hepatotoxicity of AOH degradation products was evaluated with the human fetal hepatocyte cell line L-02, known for its reliable liver function in vitro [34]. As shown in Figure 5A, the viability of L-02 cells showed a concentration-dependent decrease from 95.5% to 48.6% following exposure to 10 to 100 μΜ of AOH for 24 h. On the contrary, the treatment with 100 μΜ of crude SHP-catalyzed AOH degradation products did not significantly reduce the viability of L-02 cells (Figure 5B). The damage effect of AOH on L-02 cells was further confirmed by a significant elevation of LDH release in the culture medium of the AOH-exposed group (Figure 5C). LDH, a cytoplasmic enzyme, is swiftly discharged into the cell culture supernatant upon plasma membrane leakage, indicating cellular cytotoxicity and cytolysis. In line with the result of cell viability, there was no difference in LDH leakage between the AOH degradation products-treated group and the control group. Previous studies have revealed the key role of oxidative stress in AOH-induced cytotoxicity [35]. Consistently, AOH exposure was observed to trigger the accumulation of ROS and MDA in L-02 cells in this study, whereas the AOH degradation products did not affect cellular redox homeostasis (Figure 5D,F). To further elucidate the mechanism of oxidative stress on AOH-induced hepatotoxicity, the apoptosis rate of L-02 cells was measured by flow cytometry. The result showed that the proportion of apoptotic cells significantly increased in the AOH-exposed group in comparison with the control group, while the AOH degradation products did not promote apoptosis in L-02 cells (Figure 5G,H). In summary, crude SHP catalyzed-AOH degradation led to the detoxification of its hepatotoxic effects.

### 3.6. Performance of Crude SHP to Degrade AOH in Food Matrices

**Figure 6:**
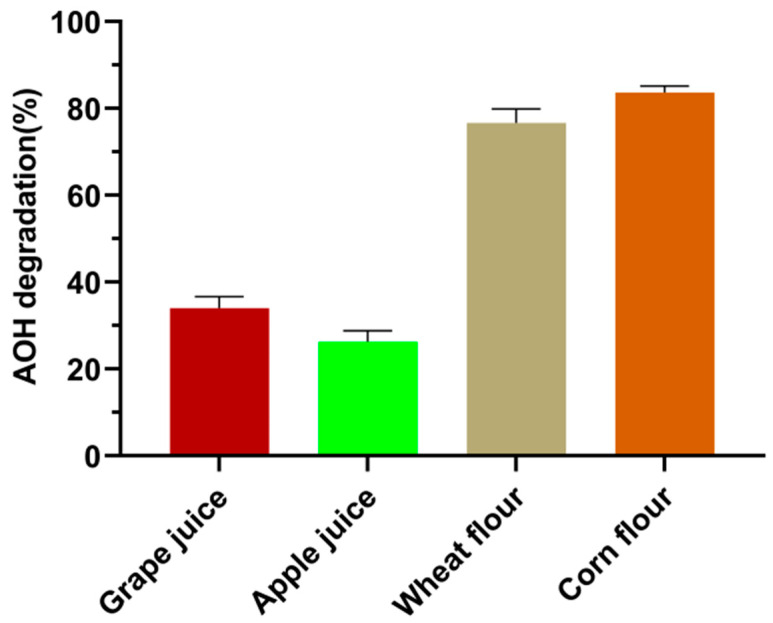
Degradation of AOH in fruit juices and cereal grains by crude SHP. Data are mean ± SD, *n* = 3.

The capacity of crude SHP to degrade AOH in buffer does not guarantee effective degradation of AOH in food matrices since the complex components might inhibit or inactivate the enzyme. The application potential of crude SHP for degradation of AOH in grape juice, apple juice, wheat flour, and corn flour were investigated in this study. The results suggested that crude SHP could achieve 77% and 84% AOH degradation in wheat flour and corn flour. In contrast, the degradation rate reduced to only 34% and 26% in grape juice and apple juice (Figure 6). The decreased AOH degradation rate in fruit juice was probably due to the low pH of grape juice (pH 3.4) and apple juice (pH 3.3), as the AOH-oxidizing activity of crude SHP was found to decline sharply at pH less than 4.0 (Figure 2A). In the literature, CotA laccase was described to degrade 18% and 22% of AOH in wheat flour and corn flour [22]. Thus, crude SHP had better catalytic efficiency for AOH degradation in cereal powder than CotA laccase. In contrast to laccase, peroxidase has an inherent shortcoming of reliance on H_2_O_2_ as a co-substrate. However, the generation of H_2_O_2_ in food products can be achieved through the use of glucose oxidase (GOD), a commonly utilized enzyme in the food industry that can catalyze the oxidation of glucose to produce H_2_O_2_. Our previous research successfully demonstrated the effective degradation of ZEN in corn steep liquor by dye-decolorizing peroxidase (DyP) in conjunction with GOD [36]. With regard to large-scale industrial application, the enzyme must be economically feasible. The direct application of crude SHP for AOH degradation in food matrices can avoid sophisticated purification procedures that are essential for the production of microbial enzymes. Thus, crude SHP is a cheap and promising choice for AOH degradation in food and feed.

## 4. Conclusions

This study examined the capacity of crude peroxidase extracted from soybean hulls to degrade AOH. The extraction process was first optimized, resulting in a maximum peroxidase activity of 36.3 U mL^−1^ achieved through extraction with 100 mM of sodium phosphate buffer at 47 °C for 4 h. Optimal degradation of AOH by crude SHP was achieved at pH 4.0–8.0, and at 42–57 °C and H_2_O_2_ concentration of 100–500 μM. UHPLC-HRMS analysis identified two AOH degradation products C_16_H_20_O_8_ and C_19_H_24_O_8_ after treatment by crude SHP. Additionally, crude SHP-catalyzed AOH degradation was found to significantly reduce its cytotoxicity to hepatocytes. Finally, crude SHP was capable of degrading 26% to 84% of AOH in tested food matrices. Overall, this study proposes the use of crude SHP as a green and sustainable enzymatic agent for AOH degradation in food and feed. However, further work is still required to fill the gap between lab-scale research and practical application.

## Figures and Tables

**Table 1:** Tentative identification of the AOH degradation products based on UHPLC-HRMS analysis.

Chemical Name	Retention Time (min)	Formula	*m*/*z*	Fragment Ions (*m*/*z*)	Inferred Chemical Structure
AOH	6.734	C_14_H_10_O_5_	257.0459	215.0385, 147.0477	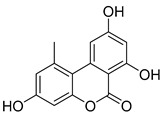
M1	11.718	C_16_H_20_O_8_	339.2319	163.1150	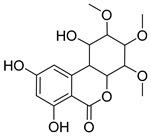
M2	12.455	C_19_H_24_O_8_	379.2289	333.2308, 275.1523	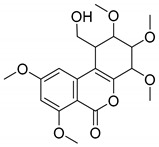

## Data Availability

The original contributions presented in the study are included in the article/Supplementary Material, further inquiries can be directed to the corresponding author.

## Supplementary Materials

The following supporting information can be downloaded at: https://www.mdpi.com/article/10.3390/foods13152434/s1, Figure S1: MS and MSMS spectra of AOH, M1 and M2.
