# Supplementary figures and images for: A Sustainable Approach for Degradation of Alternariol by Peroxidase Extracted from Soybean Hulls: Performance, Pathway, and Toxicity Evaluation
Source: Foods. 2024 Aug 1;13(15):2434. doi: 10.3390/foods13152434 (PMC11311967; doi:10.3390/foods13152434)

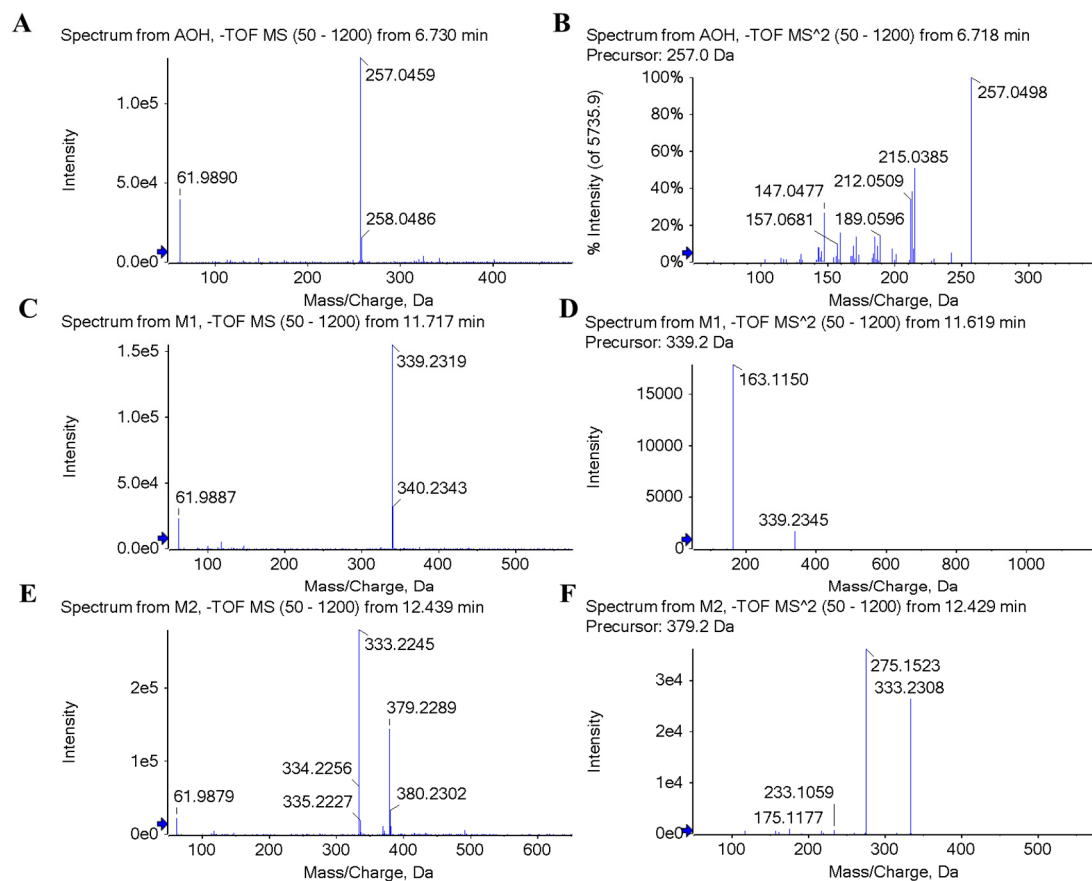

Figure S1 TOF-MS and MS/MS spectra of AOH(A-B) and degradation products (M1: C-D, and M2: E-F)

Supplement: Supplementary file 1 [file foods-13-02434-s001.zip › foods-3104543-supplementary.pdf]
